# Phylogenetic position of the ‘extinct’ Fijian coconut moth, *Levuana iridescens* (Lepidoptera: Zygaenidae)

**DOI:** 10.1371/journal.pone.0225590

**Published:** 2019-12-05

**Authors:** Vazrick Nazari, Gerhard M. Tarmann, Konstantin A. Efetov

**Affiliations:** 1 Independent Researcher, Ottawa, Ontario Canada; 2 Naturwissenschaftliche Sammlungen, Sammlungs- und Forschungszentrum der Tiroler Landesmuseen, Krajnc-Straße 1, Hall, Austria; 3 V. I. Vernadsky Crimean Federal University, Simferopol, Crimea; Museum National d'Histoire Naturelle, FRANCE

## Abstract

*Levuana iridescens* Bethune-Baker, 1906, a day-flying moth purported to be endemic to the Fijian Island of Viti Levu and a former pest of its coconut palm trees, was last observed in 1956 and has been officially declared extinct by IUCN since 1996. The controversial classical biological control method that resulted in the (presumed) demise of this moth has given this species an iconic status in biological control studies. We investigated the sister-group relationships and phylogenetic placement of this moth using NGS-obtained ancient DNA sequences from museum specimens of *L*. *iridescens* collected in the 1920s, combined with 31 morphological characters used in earlier studies and 2 new characters. Our findings show that *Levuana* is most closely related to the Australian genus *Myrtartona*. The significance of these findings is discussed.

## Introduction

The biological control program that resulted in the apparent extinction of the Fijian coconut moth, *Levuana iridescens* (Lepidoptera: Zygaenidae: Procridinae: Artonini) in the 1930s is often regarded as an example of a successful classical biological control program, or one of extinction of a native insect following the introduction of an exotic control agent [1–13]. In summary, the purposeful introduction of the Malayan parasitoid *Bessa remota* (Diptera: Tachinidae) isolated from the closely related zygaenid moth *Palmartona catoxantha* by entomologist Robert Tothill in 1925 resulted in a rapid decline and presumed extinction of the populations of *L*. *iridescens* in Viti Levu [5, 11, 14]. The moth was known only from Fiji, however, its endemicity continues to be debated [11].

In the Pacific Region, no zygaenids are known from Micronesia and Polynesia. Melanesia, on the other hand, has a rich zygaenid (Procridinae and Chalcosiinae) fauna, especially in the mountains of New Guinea. Two species are known from New Ireland: *Homophylotis aenea* Jordan, 1925 (Procridinae: Artonini) and *Heteropan lycaenoides* Walker, 1864 (Chalcosiinae: Heteropanini); one from the ‘Bismarck Archipelago’: *Homophylotis doloides* (Pagenstecher, 1900) (Procridinae: Artonini); and one from Woodlark Island: *Heteropan cyaneus* Jordan, 1925 (Chalcosiinae: Heteropanini). No zygaenids are known to occur on the islands East of Woodlark Island, except on the Solomon Islands and Fiji. Two species are known from Fiji on Viti Levu: *Levuana iridescens* (Procridinae: Artonini) and *Heteropan dolens* Druce, 1888 (Chalosiinae: Heteropanini). Tothill et al. [14] mention that they reared two ‘*Artona*’ species from larvae found on the Solomon Islands; one from Zingiberaceae, the another from banana (*Musa* sp., Musaceae). So far we have not been able to locate the material related to this rearing and therefore cannot verify these species. Banana (*Musa* sp.) is the larval hostplant of *Pseudoamuria melaleuca* (Jordan, 1908) in New Guinea [15]. Zingiberaceae are known as larval hostplants especially for species of the genus *Amuria* Staudinger, 1887.

Despite its iconic importance in biological control studies, the sister-group relationships and phylogenetic placement of *L*. *iridescens* has never been properly studied. Hering (1922) [16], Tothill et al (1930) [15] and Bryk (1936) [17] all placed it in Chalcosiinae [18]. Kalshoven (1981) [19] considered *L*. *iridescens* to be conspecific with *Palmartona catoxantha*, a widespread Indo-Malayan species (and the source host for the parasitoid, *B*. *remota*). This synonymy was subsequently questioned [7]. *Levuana* has been placed in Artonini (Zygaenidae: Procridinae) since the description of this tribe by Tarmann in 1994 [20]. In his revision of the Australian Artonini, Tarmann [15] also presented a phylogeny for all Australian genera based on 31 morphological characters, but he did not include the genus *Levuana*.

The tribe Artonini is represented in the Eastern Palaearctic, Oriental, Australian and Afrotropical regions. Diagnostic characters of this group, as described in detail previously [21], include: ***1)*** Head dorsoventrally compressed with flat occiput [15]; ***2)*** Chaetosema extending forward between the compound eye and the ocellus [15, 20]; ***3)*** A single unpaired medial spur developed on hind tibia [22–27], although it can be secondarily reduced in some species [15], this spur is absent in all species of the tribe Procridini [28–36]; ***4)*** Valva in male genitalia fan-shaped [15, 20], the dorsal and ventral sclerotisations close together when in a relaxed position but can be remarkably spread when everted from the abdominal end to hold the abdomen of the female [21]; the translucent membrane between the dorsal and ventral sclerotisations is folded; this gives the whole valva a fan-shaped appearance; ***5)*** Antenna with very movable pectinations (they can be closed to the shaft when the specimen is disturbed) [21]; and ***6)*** First instar larva with only one dorsal seta on the first abdominal segment [15, 37, 38] (plesiomorphic variant– 2 dorsal setae on the first abdominal segment).

Although the moth has been officially classified as ‘extinct’ by the World Conservation Monitoring Centre since 1996 [39], doubts have been expressed [7, 10, 11, 15, 40]. The last known specimen was collected in 1941 with further possible observational records in 1953 and 1956 [11, 41]. It has been speculated that it might still exist in low numbers on Viti Levu [10]. If the moth is rediscovered, it is likely that measures will have to be considered by the Fijian government (or international conservation organizations) towards its protection. Conservation of imperiled species, such as *L*. *iridescens*, requires correct diagnosis of their taxonomic status for effective implementation of management actions. Analysis of phylogenetic affiliations is particularly crucial in management plans that involve translocation, re-introduction, population augmentation, or captive propagation [42]. In this study we investigate the phylogenetic position of *Levuana* within the Australian Artonini using morphological characters as well as DNA barcode sequence data obtained from old museum specimens for the first time.

## Materials and methods

### Morphological data analysis

We coded thirty-one morphological characters used in a previous phylogenetic analysis of Australian Artonini [15] for *Levuana*, and added two new characters (32–33) to new data matrix (S1 File). Character 19 was coded ‘0’ for *Adscita* in Tarmann 2004 [15]; we re-coded this character as ‘2’ instead, since *A*. *statices* was the model. Only few *Adscita* species have well-developed praebursa; the spermatophore is in the praebursa in most Artonini species, but it is usually in the corpus bursae in *Adscita*.

**Fig 1:**
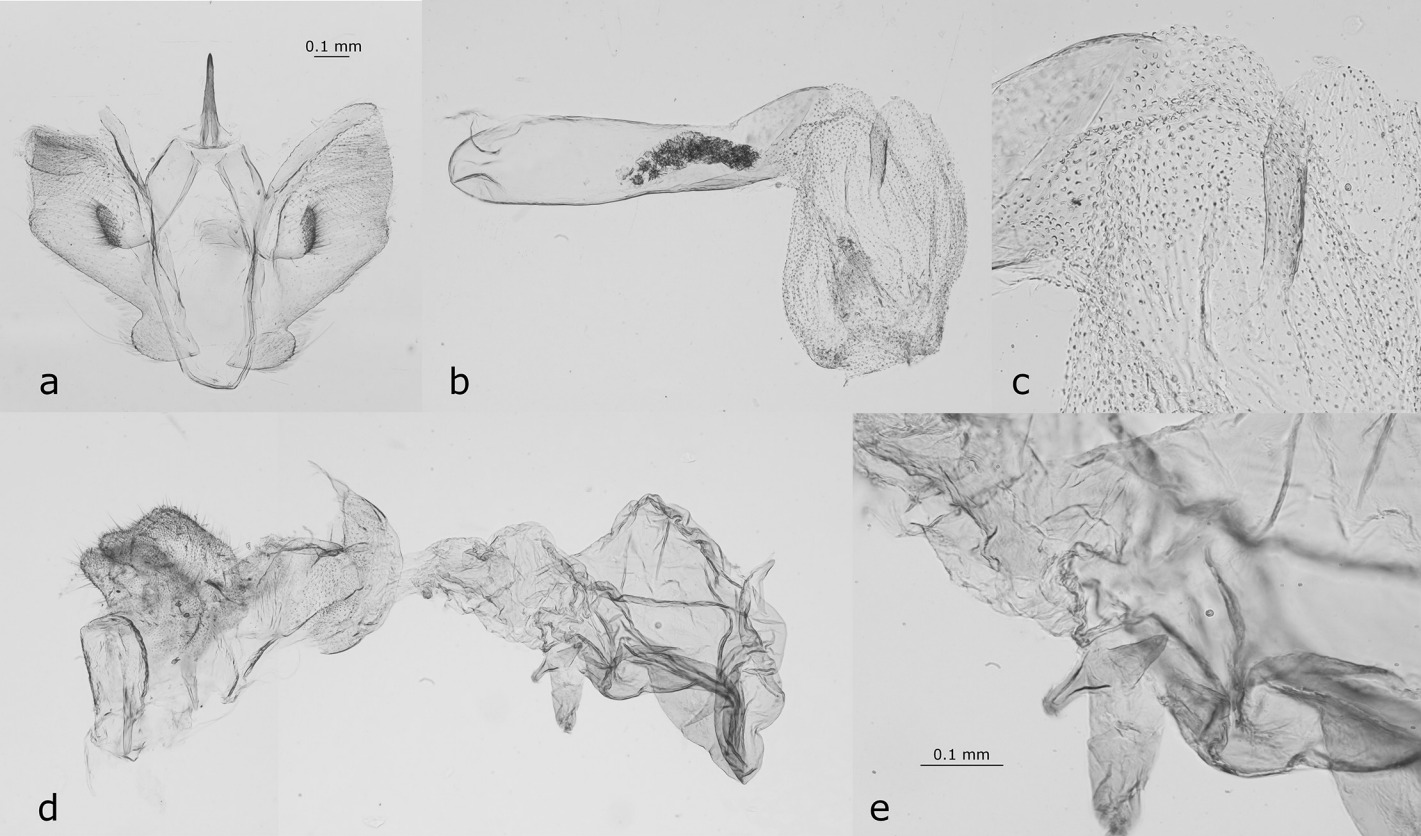
The male (a-c) and female (d-e) genitalia of *Levuana iridescens*. a) valvae, b) phallus, c) phallus (detail), d) female genitalia, e) female genitalia (detail). The scale bars for overview (a,b,d) and detail (c,e) views are the same. The female is missing the corpus bursae, and only the papillae anales, ostium, proximal part of the ductus bursae and the praebursa are visible. Dissections by Gerhard Tarmann from USNM specimens.

**Fig 2:**
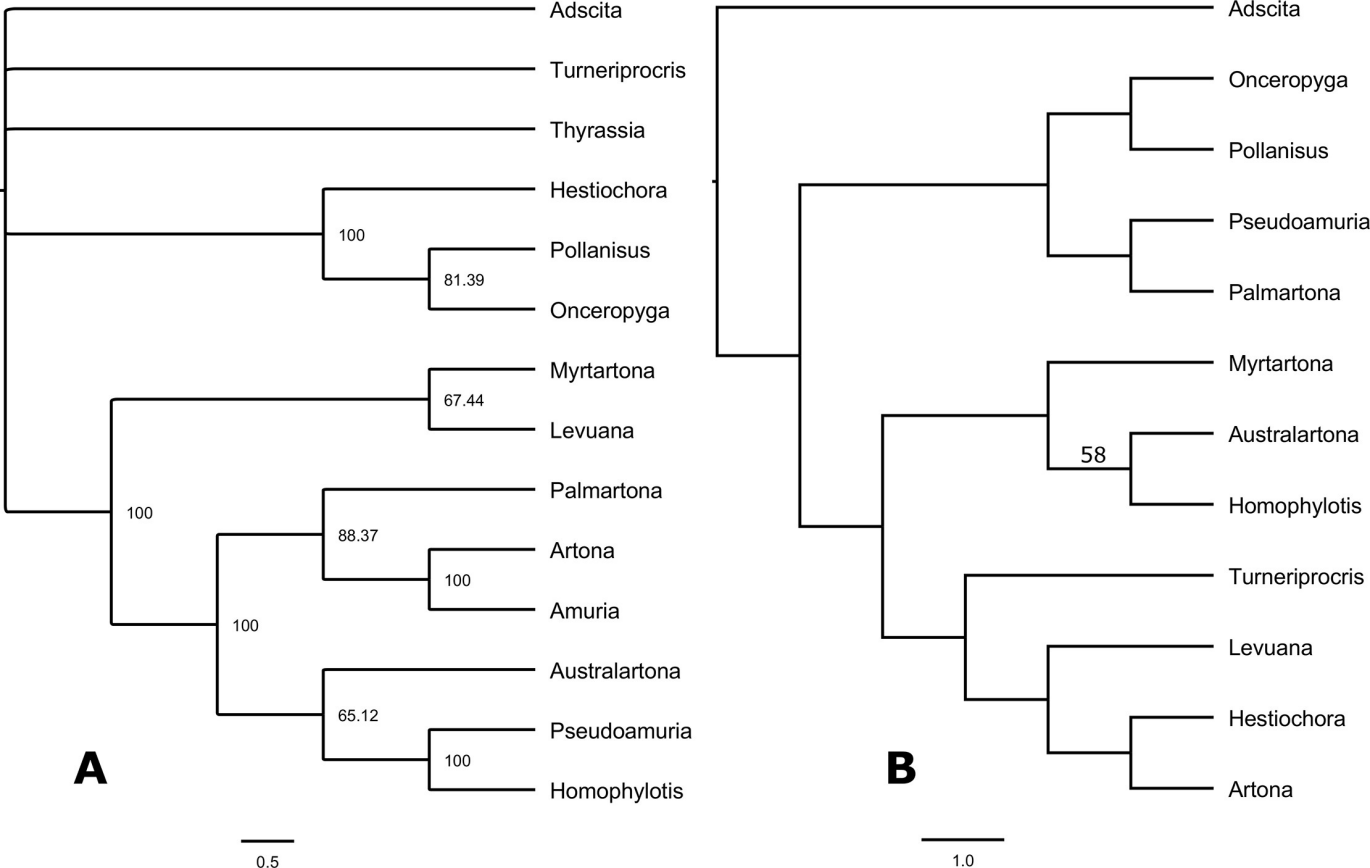
A) Tree from Branch-and-Bound analysis of morphological characters alone in PAUP. Node values indicate 50% majority rule consensus of 43 equally parsimonious trees. B) Tree from Maximum Likelihood analysis of DNA barcodes in PAUP. Only one node (*Australartona* + *Homophylotis*) has bootstrap support of over 50.

New dissections for both sexes of *Levuana* were made from USNM specimens (Fig 1), and morphological characters were coded by GMT using these specimens and dissections, as well as detailed descriptions and illustrations by Tothill et al in 1930 [14]. *Adscita* Retzius, 1783 (= *Procris* [Fabricius in Illiger], 1807), the type genus for the sister-tribe Procridini [21], was selected as outgroup. This new data matrix containing 14 taxa was analysed using PAUP* 4.0a164 [43] and a consensus tree was obtained. Branch and bound analysis yielded 43 trees of length 63, Cl = 0. 6825 (Cl excluding uninformatives = 0.6491), RI = 0.7143. The Majority Rule consensus tree is illustrated (Fig 2A).

### Molecular data analysis

In recent years, DNA barcode data for most species of Australian Procridinae have become available through Barcode of Life Campaign (v4.boldsystems.org). We selected available sequences representing the genera of Australian Artonini *sensu* Tarmann 2004 [15] for phylogenetic analysis (Table 1). Prior to this study, four relevant genera (*Amuria*, *Thyrassia*, *Palmartona* and *Levuana*) had no representation in the DNA barcode library. In aiming to complete the generic coverage, we successfully obtained sequences for two of these genera: Four specimens of *P*. *catoxantha* from Malaysia (USNM) and eight specimens of *L*. *iridescens* from Fiji (CAS: 5, UCR: 1, USNM: 2) were subjected to next-generation (NGS) sequencing. A single leg was removed from each specimen and sent to Biodiversity Institute of Ontario in Guelph, Canada for DNA extraction, amplification, and sequencing. All work was performed in a dedicated clean lab using UV sterilized equipment to minimize the risk of contamination. DNA extraction and purification was performed following the methods outlined by Prosser et al [44] and the 658 bp COI barcode region was amplified for NGS. Briefly, primers normally used for amplifying six overlapping amplicons in separate reactions were multiplexed in two PCR reactions per sample, and the resulting amplicons from each sample were pooled and sequenced on an Ion Torrent PGM (Thermo Fisher) using a 318 v2 chip and OT2 400bp chemistry (Thermo Fisher). Approximately 4.2M sequence reads were generated, which were demultiplexed into an average of 353K (±143K) reads per specimen. For each specimen, reads were processed to remove primer sequences (Cutadapt v1.8.1) and then filtered based on a minimum quality score of QV20 and a minimum length of 100bp (Sickle v1.33). The reads were then dereplicated (Fastx Toolkit v0.0.14) and assembled into a full-length barcode contig by aligning to a reference barcode sequence [45]. Final sequences were deposited in GenBank (accessions MN555769-MN555779) and all NGS reads were deposited in the Sequence Read Archive. All four *P*. *catoxantha* and only five *L*. *iridescens* specimens yielded any usable DNA sequences. All of the barcode records are also publicly available in the BOLD dataset “DS-LEVUANA”, accessed at dx.doi.org/10.5883/DS-LEVUANA. Initial NJ trees were generated using analytical modules implemented in BOLD (v4.boldsystems.org). Maximum Parsimony and Maximum Likelihood analyses were conducted in PAUP* 4.0a164 [43], and MrBayes 3.2.7 [46] was used for Bayesian analysis. Result of the Maximum Likelihood analysis is presented (Fig 2B).

**Table 1:** DNA barcoded specimens and their Genbank accessions.

Species	Sample ID	Country/Ocean	Collection Date	Length	Accession	Institution Storing
*Adscita statices*	TLMF Lep 15785	Italy: South Tyrol	2012	658[0n]	MN555775	NST
*Artona* sp.	Efetov01	Thailand	2010	658[0n]	MN555772	CSMU
*Australartona mirabilis*	10ANIC-02225	Australia: NSW	2008	658[0n]	HQ921930	ANIC
*Hestiochora erythrota*	ANIC Gen No. 001967	Australia: QLD	2005	658[0n]	MN555776	ANIC
*Homophylotis thyridota*	10ANIC-02228	Australia: QLD	1980	658[0n]	HQ921932	ANIC
*Levuana iridescens*	CASENT8406974	Fiji: Viti Levu	1920s	144[1n]	-	CAS
*Levuana iridescens*	CASENT8406973	Fiji: Viti Levu	1920s	145[0n]	-	CAS
*Levuana iridescens*	CCDB-30822-C03	Fiji	1920s	658[200n]	MN555777	USNM
*Levuana iridescens*	CCDB-30822-C02	Fiji	1920s	133[1n]	-	USNM
*Levuana iridescens*	CASENT8406976	Fiji: Viti Levu	1920s	658[200n]	MN555774	CAS
*Levuana iridescens*	CASENT8406975	Fiji: Viti Levu	1920s	325[0n]	MN555771	CAS
*Levuana iridescens*	CASENT8406972	Fiji: Viti Levu	1920s	658[250n]	MN555778	CAS
*Myrtartona rufiventris*	10ANIC-02224	Australia: W Australia	1993	658[0n]	HQ921929	ANIC
*Onceropyga anelia*	10ANIC-02192	Australia: QLD	2004	658[0n]	JF840332	ANIC
*Palmartona catoxantha*	CCDB-30822-B12	Malaysia	no date	658[200n]	MN555773	USNM
*Palmartona catoxantha*	CCDB-30822-B10	Malaysia	no date	658[89n]	MN555769	USNM
*Palmartona catoxantha*	CCDB-30822-B11	Malaysia	no date	658[95n]	MN555779	USNM
*Palmartona catoxantha*	CCDB-30822-C01	Malaysia	no date	658[0n]	MN555770	USNM
*Pollanisus viridipulverulenta*	10ANIC-02149	Australia: S Australia	2009	658[0n]	HQ921893	ANIC
*Pseudoamuria uptoni*	10ANIC-02230	Australia: QLD	1964	550[0n]	KF405409	ANIC
*Turneriprocris dolens*	10ANIC-02214	Australia: Tasmania	1991	658[0n]	HQ921926	ANIC

### Combined data analysis

**Fig 3:**
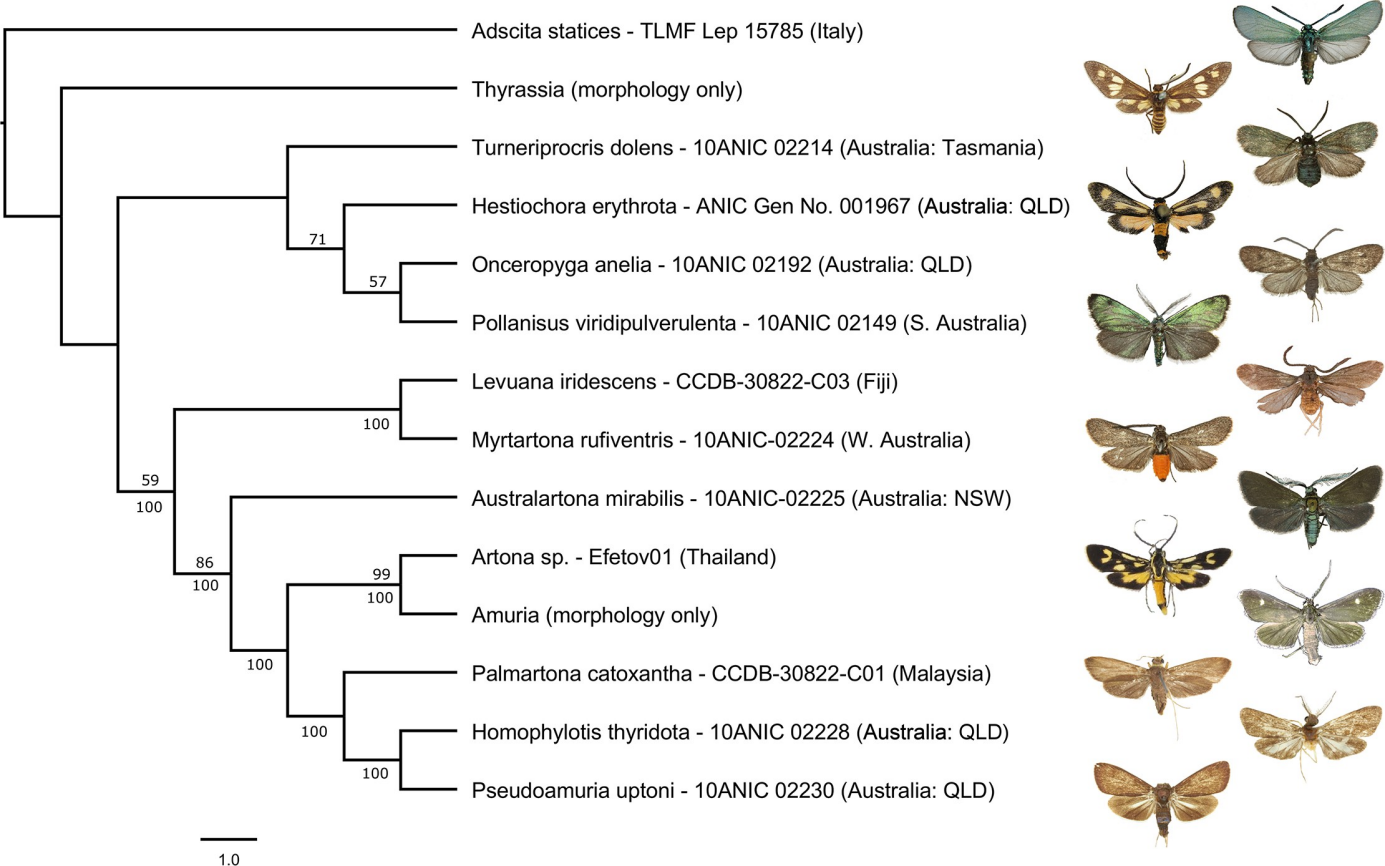
PAUP Maximum Likelihood tree from analysis of combined data (DNA + morphology). Values above branches are bootstrap support, values below branches are 50% majority rule of 5 equally parsimonious trees.

The partitioned nexus of combined data (658 bp DNA barcodes and 33 morphological characters) was initially analysed using Mesquite 3.6 [47] and repeated in PAUP* 4.0a164 [43], which yielded similar results. Add & rearrange heuristic searches were implemented for tree inference, with ‘Tree-value using Character Matrix’ and Nearest-Neighbor-Interchange (NNI) tree rearranger criteria selected. Maxtrees was set to 1000. The analysis yielded 100 trees, a 50% majority rule consensus of which is shown (Fig 3). Since no DNA sequences were available for *Amuria* and *Thyrassia*, the position of these two taxa in the phylogeny was supported only by morphological characters. Evolution of morphological characters were subsequently investigated using ‘trace character history’ function in Mesquite.

## Results

### Diagnosis of *Levuana iridescens*

A small species with elongate triangular forewing and slightly shorter, subquadrate, distally rounded hindwing. Antenna short, with symmetrical pectinations. Foreleg without epiphysis, tibial spurs 0-2-2, single medial spur on hindleg absent (present in most Artonini, secondarily lost in some species). For description of early stages, see Tothill et al [14].

### Re-description

Forewing length in male 5.5–6.2 mm, in female 6.2–7.1 mm. Head of typical artonoid form (see Tarmann 2004: 11, Figs 1–6, 59, 60), with upper part of frons and dorsal part of head capsule dark greenish blue with metallic sheen, ventral part of frons, palpi and scales around the compound eye ochreous. Frons projecting, with plate-like labrum that bears a well visible tongue-like epipharynx as a central prolongation and laterally very short, rounded pilifers, with long bristles; maxillary palps very small, one segmented; proboscis of normal length, yellow; labial palps long, pointed distally, slightly bent upwards; compound eye of medium size, black, breadth of frons almost twice as broad as breadth of compound eyes in frontal view; ocelli small, distance from the lower margin of ocellus to upper margin of compound eye ca 2.0 times the diameter of ocellus; chaetosemata small, extending forwards between compound eye and ocellus. Antenna in the male bipectinate proximally (segments 1–18), slightly biserrate distally (19–26), with a conical, pointed end-segment (27), pectinations symmetrical, sensory hairs short; female with biserrate segments 1–5 and bipectinate segments 6–18 with steadily raising length of the pectinations until segment 16 and shorter length of pectinations at segments 17 and 18, segments 19–26 slightly biserrate, with a conical, pointed end-segment (27). Thorax dark greenish blue with metallic sheen and purple tinge, legs bluish green basally, ochreous distally; dorsal abdominal segments 1 and 8 in male and 1 and 7 in female metallic greenish blue, segments 2–7 (male) and 2–6 (female) ochreous with blue tinge near middle line, ventral abdominal segments ochreous with blue tinge, especially posteriorly.

Wing venation in forewing with (R2+R3) stalked together, sometimes also together with M1, medial stem absent; hindwing with a short transverse vein between Sc and anterior margin of cell that can become vestigial.

The frenulum in *Levuana* female has only one bristle. This is a very important character that is not shared with any of the Australian Zygaenidae. *Pollanisus*, *Onceropyga*, *Hestiochora*, *Turneriprocris*, *Myrtartona*, and *Australartona* females have 3 bristles, *Homophylotis* has 2 or 3 (variable), *Pseudoamuria* and *Palmartona* have 2, and only the SE Asian genus *Thyrassia* has one bristle in both male and female. However, *Thyrassia* has many reductions and a Ctenuchid-like habitus that is very different from *Levuana*. Another important and so far unrecognized character is the distribution of scales on the wings in *Levuana*. The forewing upperside and the anterior part of the hindwing upperside until the medial stem and vein RR are densely covered with elongate triangular scales with a slightly bilobed distal end and mixed with smaller, almost needle-shaped scales that are partly arranged in a cross direction and situated below the larger scales, the posterior part of the hindwing is very translucent due to a single layer of less densely arranged upright standing needle-shaped scales. Only at the anal angle, two layers of needle-shaped scales can be found and there the hindwing is less translucent. All Australian genera and also *Palmartona* and *Thyrassia* are more densely scaled on the hindwing and are only in the central part of the hindwing sometimes more translucent.

Abdomen with lateral evaginations on segment 2 only, as in *Myrtartona*, *Turneriprocris*, *Pollanisus* and *Onceropyga*.

### Male genitalia

Uncus slender, with triangular base, short (shorter than in *Pollanisus* and *Onceropyga*), about half the length of dorsal length of valva, tapering towards and pointed at apex (as in *Turneriprocris* and not distally down-curved like in *Myrtartona*); tegumen long and slender, vinculum narrow, rounded, without ventral process; valva with fan-shaped translucent central part and a narrow sclerotisation dorsally and ventrally, without process, sacculus well-separated like in the Australian genera *Pollanisus* and *Onceropyga* (see [15]: 108, Figs 136, 136, 274), pulvinus stalked on a broad base, of similar form and in the same position as in *Pollanisus*. Phallus tube-like, approximately 5-times longer than broad, weakly sclerotized, the everted vesica of same shape as in *Pollanisus*, *Onceropyga* and *Myrtartona*, with one slender sclerotisation, somehow like the cornutus in *Pollanisus* and *Onceropyga* but shorter, without sharply pointed distal end, very similar to the structure in the vesica of *Myrtartona mariannae* (Tarmann, 2004) (see [15]: Fig 372) and a second sclerotisation that is forming an oval patch.

### Female genitalia

Very similar to that of *Pollanisus* and *Onceropyga* (see [15]: Figs 126–131 240–272, 281–282). Ostium funnel-shaped, ductus bursae not sclerotized, with a short, translucent, well-developed lateral appendix, a translucent praebursa and a small characteristic hook-like sclerotisation at the point where the ductus intrabursalis inserts into the praebursa (Fig 1E) that is constructed exactly like the ‘dagger-like structure’ in the females of *Myrtartona*, but smaller and with a short, almost triangular, pointed part and a knob-like rounder part (see [15]: Figs 374–377); ductus intrabursalis short, corpus bursae ovoid, translucent. 8^th^ sternite and tergite weakly sclerotized; papillae anales broad and well-developed, with short setae, apophyses posteriores short, apophyses anteriores extremely short, almost invisible.

### Molecular analysis

Our results support the status of *L*. *iridescens* as a distinct species with unique morphology and DNA barcode sequence that is very different from *P*. *catoxantha* (distance 10.1%). Analysis of morphological data alone revealed the position of *Levuana* in a cluster with *Myrtartona* and basal to six other genera (Fig 2A). This grouping was primarily supported by the absence of the medial stem in the venation of the forewing (character 7). The position of *Levuana* in this cluster (minus *Myrtartona*) was further supported by the monocotyledon larval hostplants (character 31).

Overall, DNA barcode distances were above 10% among the selected Procridinae (10.119%), with an average of 13.6% (Table 2). Various phylogenetic analyses of barcode sequences yielded varying topologies, as expected with phylogenies above species level that use saturated DNA barcode data alone. Support was generally low for deeper nodes, with few nodes consistently present (e.g. *Australartona + Homophylotis* and *Onceropyga + Pollanisus*) albeit with weak or no support. The position of *Levuana* was generally unstable across analyses of DNA barcode data alone. In Maximum Likelihood analyses, *Levuana* appeared in a clade with *Turneiprocris*, *Hestiochora* and *Onceropyga*, but with no support (Fig 2B). Analysis of combined data also placed *Levuana* next to *Myrtartona*, and sister to a clade containing *Australartona*, *Artona + Amuria*, and *Palmartona + Homophylotis + Pseudamuria*, with moderate support (Fig 3).

**Table 2:** Uncorrected genetic distances of COI barcodes among selected taxa. ANIC = Australian National Insect Collection; CAS = California Academy of Sciences; CSMU = Crimea State Medical University; NST = Naturmuseum Südtirol; USNM = Smithsonian Institution National Museum of Natural History.

	*Adscita statices*	*Artona* sp.	*Australartona mirabilis*	*Hestiochora erythrota*	*Homophylotis thyridota*	*Levuana iridescens*	*Myrtartona rufiventris*	*Onceropyga anelia*	*Palmartona catoxantha*	*Pollanisus viridipulverulenta*	*Pseudoamuria uptoni*	*Turneriprocris dolens*
*Adscita statices*	-											
*Artona* sp.	16.0	-										
*Australartona mirabilis*	13.5	13.9	-									
*Hestiochora erythrota*	15.4	12.8	13.7	-								
*Homophylotis thyridota*	14.1	13.2	10.1	14.3	-							
*Levuana iridescens*	13.5	12.2	11.4	11.9	11.4	-						
*Myrtartona rufiventris*	13.7	13.4	11.6	11.2	11.9	12.4	-					
*Onceropyga anelia*	16.8	17.1	17.0	13.9	16.6	16.5	15.6	-				
*Palmartona catoxantha*	12.1	13.2	11.6	12.4	10.5	10.1	12.3	13.4	-			
*Pollanisus viridipulverulenta*	12.6	15.4	13.6	12.1	15.0	13.3	13.2	14.3	12.3	-		
*Pseudoamuria uptoni*	14.9	17.9	16.3	17.8	13.8	14.5	15.1	19.0	13.3	16.8	-	
*Turneriprocris dolens*	13.4	13.0	12.8	10.5	10.5	11.4	11.1	13.4	11.7	11.7	14.0	-

## Discussion

Insects and their natural enemies, parasitoids in particular, have generally coevolved in their natural range and therefore understanding their phylogenetic affinities is crucial in biological control risk assessment. Insights into phylogenetic relationships also helps in more accurately predicting non-target hosts and assessing other risks of biological control introductions. For reasons of biosafety, host-specific parasitoids are often selected in preference over generalists, and so it is expected that closely related hosts are more likely to be at risk from attack of a parasitoid than more distantly related species. In Fiji, the introduction of *Bessa remota* to control the *L*. *iridescens* also affected *Heteropan dolens* (Druce 1888) (Zygaenidae: Chalcosiinae), another moth that suffered population decline, presumably due to parasitism by *B*. *remota* [48]. *H*. *dolens* was considered extinct until its rediscovery in 1963 [11].

The hypothesis that *Levuana* was not endemic to Fiji but probably introduced from another area of the Pacific remains unresolved, as is the native occurrence of its host, the pacific coconut *Cocos nucifera*, on Fijian coasts [49]. Lack of known records of *Levuana* prior to its first outbreak in 1877, the strictly-limited presence on Viti Levu (while most of the endemic species of Fiji are on several islands), and its later expansion into the surrounding islets support the idea that the moth was introduced to Fiji [11]. However, we find lack of records prior to its first discovery on Viti Levu in late 19th century irrelevant. Permanent winds, either from the sea to inland or vice versa, dominate most tropical islands including Fijian coasts, and only twice a day for about 30 minutes, when the winds change direction, the conditions are calm (GMT, personal observation). As far as we know, no attempts—either historical or recent—have been made to find *L*. *iridescens* in this window. These fragile little insects are most certainly only active around that time, and as in many other Artonini, it would be very difficult to encounter them only by active searching. This situation is similar in *Palmartona catoxantha*: Although this species is more common and widespread than *L*. *iridescens*, hardly anyone has observed or collected this species outside of outbreak years. Only one old specimen is known from Australia, near Brisbane (BMNH). Whether taken accidentally or a mislabeled specimen from elsewhere, this specimen was the reason for including *Palmartona* in the Australian revision of Artonini [15].

Absence of parasitoids attacking *L*. *iridescens* on Viti Levu, and thus the frequent and persistent outbreaks that regularly killed its host plants, may also suggest that *L*. *iridescens* was a late arrival to Viti Levu [11]. However, the notion that *L*. *iridescens* suddenly became a pest and had no natural enemies is not very well supported. The moth may have existed in small native colonies in its natural habitat along the coast and among the strong coastal winds, but its populations exploded only after farmers began to plant coconut trees inland and in large monocultures. Coastal winds are weak or absent on the inland, which would have created the ideal conditions for *L*. *iridescens* to develop *en masse*. Generalist parasitoids may not have been present on Fiji, explaining why none were associated with *L*. *iridescens*. Specialist parasitoids associated with other Lepidoptera, e.g. *H*. *dolens*, may occur on Fiji, although comprehensive studies are lacking. When the moth became a pest of coconut cultivations, no species-specific local parasitoids specialized on them, and thus the introduction of the non-native parasitoid (*Bessa remota*) as a biological control agent in the 1920s quickly and effectively ended the destruction of coconut palms by *L*. *iridescens* in Fiji.

The position of *Levuana* near *Myrtartona* and other Australian genera indicates that *Levuana* has likely split off from the Australian species-groups at a time before *Pollanisus* and the *Onceropyga* / *Hestiochora* group of genera had developed their striking synapomorphies (female inner genitalia without Petersen's glands but with the abdominal hair brush that covers the eggs, with presumably poisonous scales to protect the eggs). However, the genitalia characters of *Levuana* lack all the reductions of *Homophylotis* and lack also important characters from *Myrtartona* and the *Artona*-finger of *Australartona*. Alberti [50] was the first to give a differential diagnosis for the genitalia of this species in comparison with other South-East Asian and Australian Zygaenidae. He commented that the genitalia of *Levuana* are so similar to those of *Pollanisus* that he would not see a problem to treat these two taxa as two subgenera of *Pollanisus*. However, he did not change the generic status of *Levuana* because he did not have enough material for comparison from Australia and the adjacent islands to the North. The genitalia of *Levuana* seem to be intermediate between *Myrtartona* and *Pollanisus*, which indicates that *Levuana* may be of Australian (and not of Indo-Malayan) origin. Morphologically, the Indo-Malayan genera *Artona*, *Amuria*, *Palmartona* and *Pseudoamuria* are not very closely related to *Levuana*. It may well be that *Levuana* is an old relict that has been on Fiji for a long time, and is derived directly from a primitive Australian ancestor who also gave rise to *Pollanisus* and *Onceropyga* on one branch and *Myrtartona* and *Turneriprocris* on the other (see also discussion in [15]: 55). This also suggests that the hypothesis that *L*. *iridescens* is an introduced species to Fiji and originates from elsewhere is most unlikely.

Over the recent decades, the tone of discussion around the fate of *Levuana* seems to have shifted from “*a classical example of successful biological control*” [1] and “*best documented case of extinction*” [5, 8] to “*extinction of a native insect following the introduction of an exotic control agent*” [12] and to ‘*possibly not extinct at all*’ [7, 10, 11, 15, 40]. Indeed, various factors indicate that the paucity of recent records since the conclusion of *Levuana* Campaign in 1930s has more to do with lack of proper surveys than anything else [11]. The moth may still survive in Fiji in low numbers, perhaps in small inaccessible offshore islands neighbouring Viti Levu where coconut palms are not maintained for commercial use (e.g. Nukulau and Makuluva) [14]. Without the aid of artificial pheromone attractants, which are currently unknown for Artonini, the best method would be to use malaise traps on tallest palms at canopy level [11]. The best time to look for adults *of Levuana* will probably be August to December, at times of day when the winds are calm. However, even if pupal cases or cocoon clusters are found, chemical analysis of silk amino-acid composition [11], as well as DNA barcodes—published for the first time through this study—can confirm their identity.

## Supporting information

S1 File List of morphological characters used in phylogenetic analysis. For a detailed description and illustration of characters, see Tarmann 2004 [15].
(DOCX)
