# Supplementary material for: Phylogenetic position of the ‘extinct’ Fijian coconut moth, Levuana iridescens (Lepidoptera: Zygaenidae)
Source: PLoS One. 2019 Dec 5;14(12):e0225590. doi: 10.1371/journal.pone.0225590 (PMC6894762; doi:10.1371/journal.pone.0225590)
Supplement: S1 File — For a detailed description and illustration of characters, see Tarmann 2004 [15]. (DOCX) [file pone.0225590.s001.docx]

**Supplementary Information S1.** List of morphological characters used in phylogenetic analysis (For a detailed description and illustration of characters, see Tarmann 2004).

1. Presence of an epiphysis on fore tibia (0=present; 1=absent)

2. Presence of a single medial spur on the hind-tibia (0=present; 1=absent)

3. The lateral 'glands' on abdominal segments 2 and 7 in pupa and adult (0=present on abdomen on segments 2+7; 1=on segment 2 only; 2=absent)

4. Lateral evaginations enlarged on second abdominal segment in the male (0=normal; 1=enlarged in male)

5. Asymmetry in the length of the male antenna / pectinations (0=symmetrical; 1=asymmetrical)

6. The white spot on the distal part of the antenna (0=absent; 1=present)

7. Medial stem in the venation of the forewing (0=at least partly present in forewing; 1=absent)

8. Shiny, metallic scales on wings and body (0=absent; 1=present)

9. Habitus (0=not wasp-like; 1=wasp like)

10. Coloration (0=not ctenuchid like; 1=ctenuchid-like)

11. Wing pattern (0=black and yellow; 1=other)

12. Hindwing size (0=not strongly reduced; 1=strongly reduced)

13. Translucency of the hindwings (0=not translucent; 1=partly translucent)

14. The abdominal hair-tuft of the females (0=not translucent; 1=partly translucent)

15. The abdominal glands in the female (0=close to ooporus; 1=absent)

16. Female abdomen with long and slender urticating scales posteriorly (0=absent; 1=present)

17. Female with enlarged abdominal segments 7 and 8 (0=absent; 1=present)

18. Pincushion-like scales from female abdomen covering the eggs (0=absent; 1= present)

19. Prebursa in female genitalia (0=well-developed; 1=fused with corpus bursae; 2=absent)

20. Insertion of ductus intrabursalis into praebursa at special hook-like or dentate sclerotisation (0=not inserting; 1=inserting)

21. Spiny crests in tile praebursa (0=present; 1=absent)

22. Phallus with or without fixed cornutus (0=with one cornutus; 1=without cornutus; 2=with more than one cornutus)

23. Vesica with a bundle of slender, eversible (shootable) cornuti (0=present; 1=absent)

24. Vesica with large triangular or rectangular spines (0=absent; 1=present)

25. Presence of an ‘*Artona*’ -finger on the valva (0=absent; 1=present)

26. ‘*Artona*-finger’ with distal hair-brush (0=with significant distal hair-brush; 1=with a few hairs; 2=without hairs)

27. The '*Myrtartona*-finger' on the proximal part of valva (0=without finger-like dorsal connection; 1=connected with a characteristic, finger-like process dorsally)

28. Morphology of the larval thorax (0=normal; 1=with 'swollen' thorax)

29. Secondary hairs and appearance of the larva (0=without long secondary hairs and arctiid-like appearance; 1=with long secondary hairs and arctiid-like appearance)

30. Larval host-plant families (0=Myrtaceae; 1=Vitaceae; 2=Arecaceae; 3=Zingiberaceae; 4=Poaceae; 5=Dilleniaceae; 6=other)

31. Monocotyledons or dicotyledons as larval hostplants (0=monocotyledon; 1=dicotyledon)

32. Number of bristle on the female hindwing (0 = 1 bristle, 1 = 2 bristles, 2 = 3 bristles)

33. ‘Dagger-like’ sclerotisation in praebursa (with a sharply pointed part and a knob-like rounder part like a handle) (0 = absent, 1 = present)

| **Characters/Taxa** | **1** | **2** | **3** | **4** | **5** | **6** | **7** | **8** | **9** | **10** | **11** | **12** | **13** | **14** | **15** | **16** | **17** | **18** | **19** | **20** | **21** | **22** | **23** | **24** | **25** | **26** | **27** | **28** | **29** | **30** | **31** |
| --- | --- | --- | --- | --- | --- | --- | --- | --- | --- | --- | --- | --- | --- | --- | --- | --- | --- | --- | --- | --- | --- | --- | --- | --- | --- | --- | --- | --- | --- | --- | --- |
| ***Adscita*** | 1 | 1 | 2 | 0 | 0 | 0 | 0 | 1 | 0 | 0 | 1 | 0 | 0 | 0 | 0 | 0 | 0 | 0 | 0 | 0 | 1 | 0 | 1 | 0 | 0 | 2 | 0 | 0 | 0 | 6 | 1 |
| ***Artona*** | 0 | 0 | 0 | 0 | 0 | 0 | 1 | 0 | 0 | 0 | 0 | 0 | 0 | 0 | 0 | 0 | 0 | 0 | 0 | 0 | 0 | 0 | 0 | 0 | 1 | 0 | 0 | 0 | 0 | 4 | 0 |
| ***Amuria*** | 0 | 0 | 0 | 0 | 0 | 0 | 1 | 0 | 0 | 0 | 1 | 0 | 0 | 0 | 0 | 0 | 0 | 0 | 0 | 0 | 0 | 0 | 0 | 0 | 1 | 0 | 0 | 0 | 0 | 3 | 0 |
| ***Palmartona*** | 0 | 0 | 0 | 0 | 1 | 0 | 1 | 0 | 0 | 0 | 1 | 0 | 0 | 0 | 0 | 0 | 0 | 0 | 0 | 0 | 0 | 2 | 1 | 0 | 1 | 2 | 0 | 1 | 0 | 2 | 0 |
| ***Pseudoamuria*** | 0 | 1 | 0 | 0 | 0 | 1 | 1 | 0 | 0 | 0 | 1 | 0 | 0 | 0 | 0 | 0 | 0 | 0 | 0 | 0 | 0 | 1 | 0 | 0 | 1 | 1 | 0 | ? | 0 | ? | 0 |
| ***Homophylotis*** | 0 | 1 | 0 | 1 | 1 | 1 | 1 | 0 | 0 | 0 | 1 | 0 | 1 | 0 | 0 | 0 | 0 | 0 | 1 | 0 | 1 | 1 | 1 | 1 | 1 | 1 | 0 | ? | 0 | ? | 0 |
| ***Australartona*** | 0 | 1 | 0 | 1 | 0 | 0 | 1 | 0 | 0 | 0 | 1 | 0 | 0 | 0 | 0 | 0 | 0 | 0 | 2 | 0 | 1 | 1 | 1 | 0 | 1 | 2 | 0 | ? | 0 | ? | 0 |
| ***Myrtartona*** | 1 | 1 | 1 | 0 | 0 | 0 | 1 | 0 | 0 | 0 | 1 | 0 | 0 | 0 | 0 | 0 | 0 | 0 | 0 | 1 | 1 | 0 | 1 | 0 | 0 | 2 | 1 | 0 | 0 | 0 | 1 |
| ***Turneriprocris*** | 1 | 1 | 1 | 0 | 0 | 0 | 0 | 0 | 0 | 0 | 1 | 0 | 0 | 0 | 0 | 0 | 0 | 0 | 2 | 0 | 1 | 1 | 1 | 0 | 0 | 2 | 1 | 0 | 0 | 0 | 1 |
| ***Pollanisus*** | 1 | 1 | 1 | 0 | 0 | 0 | 0 | 1 | 0 | 0 | 1 | 0 | 0 | 1 | 1 | 1 | 1 | 1 | 0 | 1 | 1 | 0 | 1 | 0 | 0 | 2 | 0 | 0 | 0 | 5 | 1 |
| ***Onceropyga*** | 1 | 1 | 1 | 0 | 0 | 0 | 0 | 0 | 0 | 0 | 1 | 0 | 0 | 1 | 1 | 1 | 1 | 1 | 0 | 0 | 1 | 0 | 1 | 0 | 0 | 2 | 0 | 0 | 0 | 1 | 1 |
| ***Hestiochroa*** | 1 | 1 | 2 | 0 | 0 | 0 | 0 | 0 | 1 | 0 | 1 | 0 | 0 | 1 | 1 | 1 | 1 | 1 | 2 | 0 | 1 | 1 | 1 | 0 | 0 | 2 | 0 | 0 | 0 | 0 | 1 |
| ***Thyrassia*** | 1 | 1 | 2 | 0 | 0 | 0 | 0 | 0 | 0 | 1 | 0 | 1 | 0 | 0 | ? | 0 | 0 | 0 | 2 | 0 | 1 | 1 | 1 | 0 | 0 | 2 | 0 | 0 | 1 | 1 | 1 |
| ***Levuana*** | 1 | 1 | 2 | ? | 0 | 0 | 0 | 1 | 0 | 0 | 1 | 0 | 0 | 0 | 1 | 0 | 0 | 0 | 0 | 0 | 1 | 0 | 1 | 0 | 0 | ? | 0 | 0 | 0 | 6 | 1 |
